# Are Accuracy and Reaction Time Affected via Different Processes?

**DOI:** 10.1371/journal.pone.0080222

**Published:** 2013-11-18

**Authors:** Martijn J. Mulder, Leendert van Maanen

**Affiliations:** 1 Cognitive Science Center Amsterdam, University of Amsterdam, Amsterdam, The Netherlands; 2 Department of Psychological Methods, University of Amsterdam, Amsterdam, The Netherlands; McMaster University, Canada

## Abstract

A recent study by van Ede et al. (2012) shows that the accuracy and reaction time in humans of tactile perceptual decisions are affected by an attentional cue via distinct cognitive and neural processes. These results are controversial as they undermine the notion that accuracy and reaction time are influenced by the same latent process that underlie the decision process. Typically, accumulation-to-bound models (like the drift diffusion model) can explain variability in both accuracy and reaction time by a change of a single parameter. To elaborate the findings of van Ede et al., we fitted the drift diffusion model to their behavioral data. Results show that both changes in accuracy and reaction time can be partly explained by an increase in the accumulation of sensory evidence (drift rate). In addition, a change in non-decision time is necessary to account for reaction time changes as well. These results provide a subtle explanation of how the underlying dynamics of the decision process might give rise to differences in both the speed and accuracy of perceptual tactile decisions. Furthermore, our analyses highlight the importance of applying a model-based approach, as the observed changes in the model parameters might be ecologically more valid, since they have an intuitive relationship with the neuronal processes underlying perceptual decision making.

## Introduction

**Figure 1:**
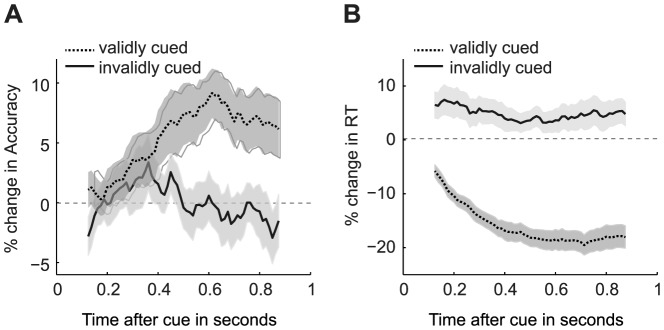
Behavioral time courses showing the effects of a valid or invalid attentional cue on accuracy and the response time (RT) of tactile decisions. Lines represent the average accuracy (**A**) and the average RT (**B**) for validly (solid) and invalidly (dashed) cued trials within each temporal window.

In perceptual decision making, attention has been shown to affect choice behavior [1]–[4]. How attentional cues are processed by the brain to affect decision process remains an open question. Recently, van Ede *et al*., (2012) [5] addressed this question by investigating whether the validity of an attentional cue affects the accuracy and speed of a tactile perceptual choice via different cognitive and neural processes. In their experiment, participants received an auditory cue that indicated if a tactile stimulation would be applied to the left or to the right hand. To investigate the temporal dynamics of prior knowledge on the decision process, the authors manipulated the time between a cue and the following tactile target. Time courses were calculated for reaction time (RT) and accuracy using a moving-window approach across different cue-target-intervals (CTI; see Figure 1).

**Figure 2:**
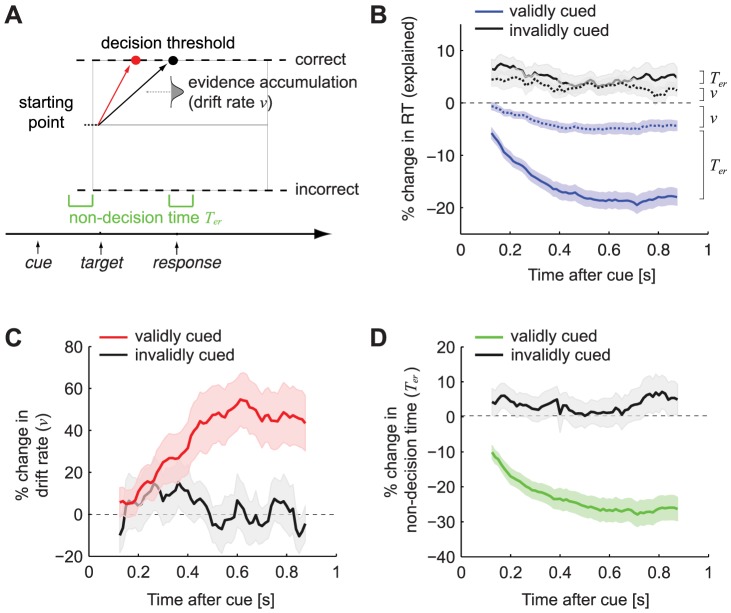
Results from fitting the DDM to the data of van Ede *et al*. **A**. The drift-diffusion model (DDM) assumes an accumulation process, until the evidence reaches a decision threshold. An increase of drift rate causes faster and more correct choices (red arrow). Non-decision time reflects the time other then the decision time (e.g. process sensory information and execute a motor response). **B**. Change in reaction time (RT) explained by drift rate (*v*) and non-decision time (*T_er_*) for validly (blue) and invalidly (grey) cued trials compared to the baseline (uncued) RT. **C**. Time course for drift rate (*v*), showing the percentage increase from the baseline drift rate for validly (red) and invalidly (grey) cued trials. **D**. Time course for the percentage change in non-decision time (*T_er_*) for validly (green) and invalidly (grey) cued trials compared to the baseline non-decision time.

Importantly, van Ede *et al.* obtained magnetoencephalography (MEG) recordings during the tactile paradigm. These MEG recordings were found to explain the time course of the increasing accuracy over CTI, but not the time course of the decreasing RT over CTI. Based on these findings, the authors proposed a model where changes in accuracy are explained by an increase of a preparatory signal in the sensory cortex, whereas changes in RT are explained by an additional process required to compare the expected and the actual stimulus [5]. These results suggest that accuracy and RT are affected by attention via different neural and cognitive processes. Although interesting, the conclusions are remarkable, as they undermine the notion of a close coupling between accuracy and RT that has been advocated by many [6]–[10]. In particular, in perceptual decision making, mathematical models are used to describe and predict changes in the dynamics of the decision process. Such models, like the drift diffusion model (DDM), conceptualize the decision process as the accumulation of sensory information over time toward a decision threshold (Figure 2A; for review see [7]). Typically, these models can explain variability in both speed and accuracy by a single parameter. Along these lines, it might be the case that the observed MEG pattern reflects a change of a single DDM parameter, which can explain both accuracy and RT effects. This would suggest that the cognitive processes underlying accuracy and RT are not so separate after all.

To investigate which processes drive the behavioral results that were reported by van Ede et al., (2012), we fit the DDM to both the accuracy and RT data. We hypothesize that the accuracy and RT time courses can be described by a gradually increasing drift rate (v), as a function of an increasing CTI. Additionally, we hypothesize that the perception of the cue might interfere with the perception of the target, which might change the encoding of sensory information before the actual decision is made (cf. the PRP paradigm, [11]). Such an effect will typically affect the non-decision time (T_er_; [12]). Results will uncover possible latent processes that might drive the observed effects of the attentional cue in accuracy and RT, as reported by van Ede et al. (2012).

## Materials and Methods

Below we will first describe in short the task used by van Ede et al., (2012). For details we refer to their original paper. Next, we will describe the methods used to fit the drift-diffusion model to the data.

### Task

Seventeen participants performed two sessions (∼1500 trials) of a cued somatosensory task in which they where asked to decide whether a tactile stimulus of 20 ms was presented at the lower or upper part of the fingertips of either the left or right hand. In 80% of the trials, the stimulus was preceded by an auditory cue of 25 ms that indicated at which hand the tactile stimulus could be expected. In 75% of these cued trials, the cue was valid, meaning that the stimulus occurred at the expected hand. In 25% of the cued trials, the stimulus occurred at the unexpected hand (ie., the cue was invalid). Importantly, the cue-stimulus-interval (CTI) varied across the cued trials: At each trial, a new CTI was drawn randomly from a uniform distribution ranging from 0 to 1000 ms. These different CTI's allowed for a time course analyses of the cue effects.

### DDM Expectations

Typically, a model-based explanation of both an increase in accuracy and a decrease in RT involves a change in either the starting point (e.g. [13]–[19]), or the drift-rate of the decision process (e.g. [13], [17], [20], [21]). However, the task used by van Ede et al., (2012) was balanced in such a way that the attentional cue was not informative for the different choice alternatives. That is, the cue represents prior information about the likelihood that the stimulus would be applied to the left or to the right hand and not about the choice alternatives (upper or lower part of the fingertips). As such, by design, the differences in validly and invalidly cued trials can not be the result of a starting point difference.

In contrast, an increase in the drift rate would result in faster and more correct choices for the cued alternative [1]–[4], which is in line with the observed behavioral results. As such, we expect that the main effects of the attentional cue in accuracy and RT can be explained by a change in drift rate. However, other parameters might be involved as well. For example, since the task design is as such that the attentional cue will affect both bounds, it might affect the boundary separation (decision threshold) parameter of the model. Additionally, as short CTI's might result in sensory interference [12], we might expect non-decision time effects in RT as well. Note, however, that these parameters are unlikely to explain the data in isolation: the non-decision time parameter cannot explain the observed differences in accuracy and a change in the boundary separation parameter can only predict an increase in accuracy when slower response times are expected (speed-accuracy trade off). For this reason, we tested whether these parameters play a role in the decision process, in combination with the drift rate parameter.

### DDM model-selection

To test which of these parameters are required to explain the behavioral effects, we first run a model-selection procedure using the whole dataset (that is, not split for different CTIs). Parameters of four models were estimated: Model 1, in which drift rate was allowed to vary across the three conditions (valid, invalid, uncued); Model 2, in which both drift rate and non-decision time were allowed to vary across the three conditions; Model 3, in which both drift rate and boundary separation were allowed to vary across conditions; and Model 4, in which drift rate, non-decision time and boundary separation were all allowed to vary across the three conditions. All other parameters were held fixed across conditions. We used the Diffusion Model Analysis Toolbox (DMAT) to fit the DDM to the individual data [22]. The DMAT toolbox maximizes the likelihood of observing a proportion of responses within a given number of reaction time (RT) bins (the 0.1, 0.3, 0.5, 0.7, 0.9 quantiles) using SIMPLEX optimization routines [23]. Because the fit of more complex models (with more free parameters) is necessarily better than (or equally good as) the fit of simpler models, it is crucial to consider the number of free parameters when performing model comparisons. Therefore, for each subject and each model, the Bayesian information criterion (BIC) was calculated to determine the model with the best trade-off between fit quality and model complexity [24], [25]. The BIC corrects the log-likelihood of the model's fit to the data based on the number of free parameters, in such a way that if a more complex model is only slightly better in terms of fit, the simpler model will have a better (lower) BIC value, and should be preferred. To compare between the BIC values of the different models, we calculated for each model and each subject the BIC weights [24]. These BIC weights represent the probability that the model is the best model, compared to all other fitted models. The BIC weights were averaged across all subjects. Next, the model with the largest average BIC weight was chosen as the model that explained the difference between conditions (valid/invalid and neutral) the best. This model was then fitted to the data of each separate time-window to obtain the parameter time-courses.

### DDM time courses

We fitted the best model to both the accuracy and RT data of the valid and invalid conditions using a moving window approach. Each window was defined as a CTI range of 250 ms that moved in 60 steps from 125 to 875 ms (see [5]). The model-parameters that accounted for the difference in accuracy and RT between the cued and uncued conditions in the whole dataset (see above) were free to vary across the valid and invalid conditions of each window. This allowed us to determine *how* these parameters were affected by the validity of the attentional cue, over the course of the increasing CTI.

### Post-hoc analysis of percentage change from baseline

To test whether the model parameters changed with CTI, we performed a 2×2 repeated measures ANOVA with window and validity as within subjects factors. To diminish the dependencies between parameters due to the moving-window approach, we only considered the first (from 0 ms to 250 ms) and the last window (from 750 ms to 1000 ms).

## Results

The study by van Ede et al., (2012) shows that the accuracy and reaction time (RT) of tactile perceptual decisions are affected by the validity of an attentional cue via different cognitive and neural processes. We fitted the drift diffusion model to their data to decompose the decision process into parameters that represent latent cognitive processes that underlie the tactile choices.

**Figure 3:**
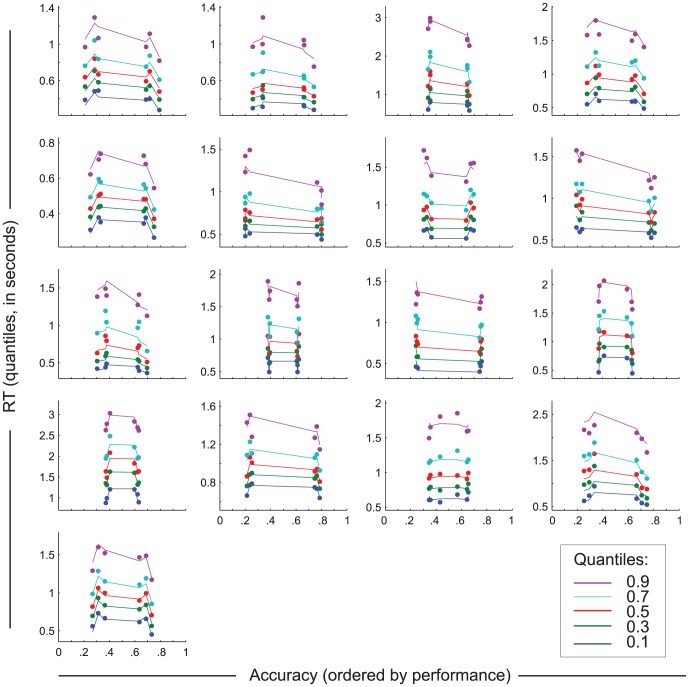
Quantile probability plots showing the best fitting model (see methods) for each subject. Each graph represents the proportion correct choices and reaction time (RT) distributions for each condition (data points) and the DDM quantile probability functions describing them (lines). RT distributions are represented by five quantiles (colors), plotted along the y-axis for each condition. Conditions (neutral, validly and invalidly cued trials) are split into correct and incorrect responses and divided over the x-axis, representing response probability. Lines connecting the quantiles between conditions represent changes in RT distributions across conditions, for incorrect and correct responses.

For each subject we first fitted the DDM to the whole data set and performed model selection. For sixteen of the seventeen subjects, we found higher BIC weights [24] for Model 2: The model with both a variable drift rate and non-decision time was more likely to explain the accuracy and RT data than the other models (mean[SD] BIC weights for Model 1 = 0.06[0.24], Model 2 = 0.67[0.40], Model 3 = 0.14[0.31] and Model 4 = 0.14 [0.25]). Figure 3 shows, for each subject separately, the accuracy and RTs for the experimental conditions across the whole data set, together with the accompanying predictions of Model 2.

**Figure 4:**
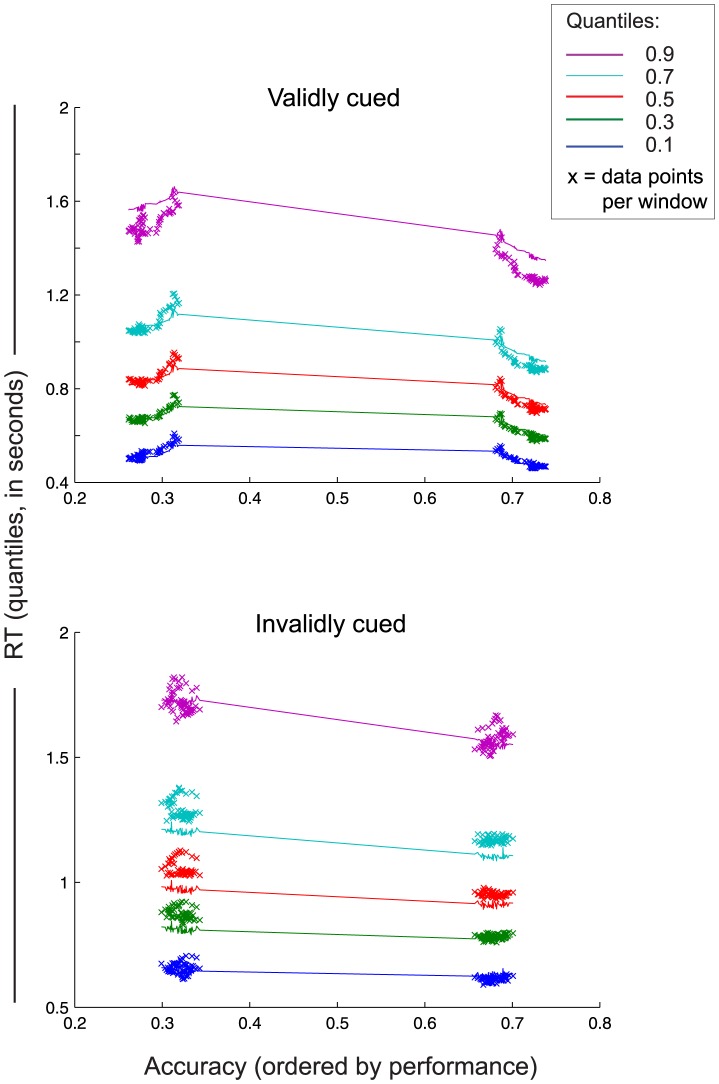
Group quantile probability plots for the validly and invalidly cued trials. Data points represent group mean for correct (right) and incorrect (left) choices in each time course window. Lines represent the group average of the DDM predicitions.

To obtain the parameter time courses, we fitted Model 2 to the data using a moving window approach (see Materials and Methods). For each window, we estimated the drift rate (*v*) for validly and invalidly cued trials, which is thought to reflect the quality of accumulated evidence (Figure 2A). In addition, we estimated a non-decision component (non-decision time *T_er_*) for validly and invalidly cued trials reflecting changes in sensory and motor processes [7]. All other parameters where held fixed for each window using the participant-specific parameter values that were estimated by fitting the DDM on the whole dataset. Figure 4 shows the quantile probability plots (QPP) for each window, averaged over participants, separately for the validly and invalidly cued conditions. With the constraint in mind that only drift rate and non-decision time were allowed to vary across conditions the model fits are reasonably good. There is some deviation between the data and the model in the tails of the distribution. This is a common observation, often explained by the increased spread in the tails of RT distributions. Note also that, for the invalidly cued trials, the predicted RTs are faster, resulting in a deviance between the model and the data. This is especially visible for the middle (0.3, 0.5 and 0.7) quantiles. This deviance is most likely a reflection of the lower number of trials for the invalidly cued trials (mean(SD) #trials  = 85.8 (34.9) for invalid, against 254.6 (110.8) for validly cued trials), resulting in a larger variance in the RT data, both within and across subjects.

To show how the model parameters change over the increasing CTI, a time course for the drift rate and non-decision time was constructed by calculating the percentage change from their baseline values within each subset of the data.

Results show that changes in accuracy and RT can be explained by a combination of processes: for validly cued trials, we found an increase in drift rate and a decrease in non-decision time when CTI increases (Figure 2C, D; interaction validity × window resp. F(1,16) = 7.6; p = 0.014 and F(1,16) = 19; p<0.001). Figure 2B shows the RT timecourse separated in RT changes related to drift rate (*v*) and RT changes related to non-decision time (*T_er_*). These results confirm that both drift rate and non-decision time are necessary to explain a substantial part of the RT data. Note however that, although there is no difference in drift rate between the validly and invalidly cued conditions at the very early CTIs (see Figure 2C), we do find a difference between the RT effects related to drift rate for these conditions at these time points (see Figure 2B). This unexpected effect is most likely due to the larger variance in the RT data of invalidly cued trials resulting in an overestimation of the drift rates for invalidly cued trials (see also Figure 4). As a result, the real drift rate for invalidly cued trials might be lower than the model predicts, explaining the early difference between RT effects between baseline and invalidly cued trials.

## Discussion

A recent study by van Ede et al. (2012) shows that the accuracy and reaction time (RT) of tactile perceptual decisions are affected by the validity of an attentional cue via distinct cognitive and neural processes. To elaborate this finding, we fitted the drift diffusion model to the data. The observed changes in the DDM parameters provides a subtle explanation of how the speed and accuracy of cued tactile decisions are affected by two distinct processes, as argued by van Ede *et al*. The increase in accuracy for validly cued trials can be explained by a gradual increase in drift rate. The decrease in RT for validly cued trials can be explained by this increase in drift rate as well, but only partly: an additional decrease of the non-decision time is necessary to account for effects in RT that are unrelated to the decision process (e.g. sensory or motor processes; Figure 2B, D).

Note also that the model with variable drift rate and non-decision time produced a better fit to the data than the model where only drift rate was allowed to vary across conditions (as measured by a better balance between fit quality and model complexity [25]). As such, the observed behavioral changes seem to be driven by two distinct processes. However, these processes do not separate the accuracy and RT effects in the same way that van Ede et al., (2012) suggested by their MEG analysis. In this analysis, the authors transformed the behavioral and MEG time-courses using a logistic function. Results of these transformations showed that the time-course of the MEG signal was similar to the time-course of the increase in accuracy (for validly vs invalidly cued trials), leading to the conclusion that the MEG signal could only explain the observed accuracy, but not the observed RT effects. As such, the authors concluded that the attentional cue affects accuracy and RT via different cognitive and neuronal processes. However, our results show that the time-course of the changing drift rate is remarkably similar to the effects observed in the accuracy time-course (see Figure 1A, and 2C). Therefore, it is likely that the reported MEG signal by van Ede et al. (2012) drives the change in drift rate, and not solely the change in accuracy. In addition, drift rate affects RT as well, which is shown by Figure 2B. As such, both changes in drift rate and non-decision time are necessary to account for the effects of the validity of the attentional cue observed in the RT time course.

In sum, by fitting the DDM to the data of van Ede et al, we were able to distinguish between processes that are related to a decision component (drift rate) and a process related to a sensory or motor component (non-decision time). Both of these processes affect RT (drift rate & non-decision time) with one of them (drift rate) simultaneously affects accuracy as well.

Another advantage of using a formal model in the behavioral analysis is that it allows us to interpret the behavioral results of the study by van Ede *et al*. within a conceptual framework that describes the dynamics of the decision process. Drift rate is thought to reflect the quality of sensory evidence [7], which seem to improve by attentional cueing. This effect in drift rate might be compatible to the proposed model by van Ede *et al*. where an increase of the preparatory signal in the sensory cortex results in an increase of the level of performance. Furthermore, the compatibility effect described by the authors might influence RT via the non-decision component where the validly cued choices benefit from the cue prior to the target, resulting in a faster cue-target comparison [5]. However, the authors note that in their proposed model these different effects on RT cannot be disambiguated, and as a consequence it will be problematic to infer preparatory processes solely on the basis of RT data. By using a model-based approach we are able to disentangle the RT effects in two measurable parameters that might be used to identify the underlying neuronal processes that a responsible for both accuracy and the different RT effects.

**Figure 5:**
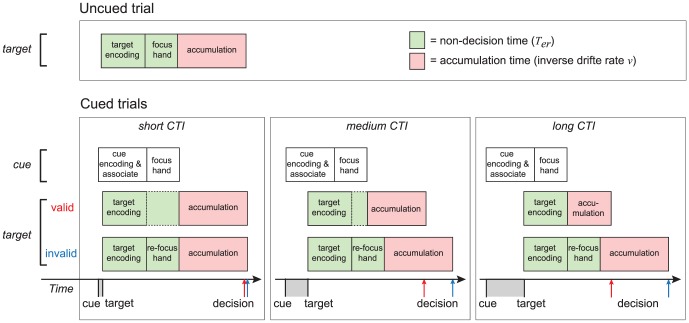
Effects of the validity of an attentional cue on non-decision time and drift rate result in changes in accuracy and RT. For uncued trials, the process of accumulating tactile evidence (red; drift rate) is preceded by the encoding of the target and an attentional focus to the stimulated hand (green; non-decision time). For the cued trials, the encoding of the auditory cue is followed by a focused attention to the hand associated with the cue (white boxes). A target may benefit from this process when the cue is valid and the cue stimulus interval (CTI) is long enough, as the attention is already drawn to the stimulated hand. This results in shorter non-decision times and improved processing of sensory information, which in turn will lead to shorter accumulation times due to an increase in drift rate. For short CTIs, this advantage is minimal, as the processing of the auditory cue results in a delay in processing the target, resulting in cognitive slack time (empty green boxes). In contrast, invalidly cued trials lack the advantage of focused attention to the relevant hand. The onset of the target results in a re-focus of the attention to the other hand. As such, drift rate and non-decision time will be similar to those observed in the uncued trials, across different CTIs. These processes lead to divergence in decision times for validly cued and invalidly cued trials, as indicated on the timeline with red (valid) and blue (invalid) lines.

An alternative explanation of the observed effects of the validity of the attentional cue might involve the parallel processing of the attentional cue and the tactile target (Figure 5). According to this idea, the encoding of the auditory cue is followed by a focused attention to the hand associated with the cue. For short CTIs, this process results in a delay in processing the target, resulting in a psychological refractory period (PRP; [11], [12], [26], [27]). To understand the similarity with a typical PRP paradigm one could think of the tactile task used by van Ede et al. (2012) as two separate tasks that the participants had to perform simultaneously. *Task 1* involves detection of the cue, which automatically draws the attention to the cued hand by means of a stimulus-response association (see [28]). *Task 2* involves target detection, which draws the focus to the stimulated hand. Although Task 2 might benefit from a valid cue in Task 1 when the focus of attention is similar for both tasks, at very small CTIs the processing of a target in Task 2 will still be delayed by the processing of the cue itself (Task 1). This effect becomes smaller for larger CTIs, as the overlap between the tasks becomes less. However, when the cue is invalid, the benefit is not apparent, as the target always triggers a refocus to the other hand after an invalid cue.

This PRP-like effect might account for both the increase in non-decision time and the decrease in drift rate: processing the cue causes a delay in processing the target (non-decision time) and interferes with the process of collecting tactile evidence of the spatial location of the target (drift rate; see Figure 5). As explained, when CTI increases, the influence of the PRP effect gradually becomes less apparent. As such, non-decision time decreases, and drift rate increases (resulting in a faster accumulation time). However, for the invalidly cued trials, the drift rate and non-decision time will be similar to those observed in the uncued trials, across different CTI's (see Figure 5). In all, this alternative explanation explains how the validity of an attentional cue affects the dynamics of the decision process, resulting in the observed behavioral effects. Furthermore, the model-based approach provides a framework where different components of the decision process can be measured, which might be useful in investigating the underlying neuronal processes of tactile decision making [29].

Importantly, although a model like the DDM might be informative at the cognitive level and provide explanations that are biologically plausible, the dynamics of the underlying neural network may be more complex then the model predictions assume (e.g., [30], [31]) That is, subtle changes in the underlying neuronal network can result in mechanistically different predictions than would have been expected from a model-based account (e.g., [32]). For example, the DDM cannot distinguish between different mechanisms *within* the non-decision time that might affect the decision process [33]. As such, although the model-based approach is useful to inform the analyses of brain imaging methods, and to identify latent cognitive processes, caution has to be made in suggesting a one-on-one mapping from the model onto the neural substrate underlying the decision process.

Taken together, van Ede *et al*. show that the speed and accuracy of tactile decisions have differentiating time courses depending on the validity of an attentional cue. The model-based approach provides a subtle explanation of how the underlying dynamics of the decision process might give rise to these different effects. In particular, we show here that the validity of the attentional cue affects the decision making process, but that the temporal proximity of a cue might interfere with general processing of a subsequently presented target stimulus. Our analyses highlight the importance of applying a model-based approach, as it shows that the underlying processes of accuracy and RT might not be so distinct after all. Furthermore, the observed changes in the estimated model parameters might be ecologically more valid, as they have a intuitive relationship with the neuronal processes underlying perceptual decision making [29], [34], [35].
